
JOURNAL INFORMATION
==============================
NLM Title Abbreviation: Tex Med J (Austin)
NLM Journal ID: 21130590R

Texas Medical Journal

ISSN: 0892-8495
Publisher: Biomedical Journal Digitization Project

ARTICLE INFORMATION
==============================
PMCID: PMC9595846
Article version: 1
Subjects: Books and Magazines

A Manual of Venereal Diseases

Print publication date: 1899 Dec
Volume: 15
Issue: 6
First page: 347
Last page: 347
License: This work is free of known copyright restrictions. For more information, please see PMC Back Issue Digitization.
License URL: https://creativecommons.org/publicdomain/mark/1.0/

==============================

work. Nearly the entire book is devoted to laboratory experiments, there being about two hundred in all. As a thoroughly practical guide for students there seems to be nothing lacking. B.

---

A MANUAL OF VENEREAL DISEASES. By James R. Hayden, M. D., Chief of Clinic and Instructor in Genito-Urinary and Venereal Diseases, College of Physicians and Surgeons, New York; Professor of Genito-Urinary and Venereal Diseases in the Medical Department of the University of Vermont, etc. New (2nd) edition, revised and enlarged. In one 12mo. volume of 304 pages, with fifty-four engravings. Cloth, $1.50, net. Lea Brothers & Co., publishers, Philadelphia and New York.

This book contains about 300 pages, is well arranged as to subject matter and has a great many illustrations. It is devoted strictly to the three venereal diseases, gonorrhœa, chancroid and syphillis and their immediate complications. If the busy doctor, who has not in a long time stopped and read a book on this subject, will take the time to read Hayden's Manual, he will find several difficulties in venereal practice easily overcome. It will pay him to put the book in his library for reference. B.

---

### Clinical Reports.

### Irregular Menstruation in Young Women Due to Anaemic Conditions.

BY H. EDWIN LEWIS, M. D., BURLINGTON, VT.
Resident Physician Fanny Allen Hospital.

The young physician just starting into practice cannot help but be impressed with the frequent occurrence of menstrual disorders in young girls during the period just succeeding the age of puberty. The metamorphosis of a girl into a woman, consisting as it does of structural and functional changes throughout her body, in many instances leaves behind pronounced alterations in the quality or even quantity of the blood current. How common it is to have a mother bring her daughter to the physician and say, "Doctor, I would like to have you do something for my daughter. For nearly a year she has been losing interest in everything and seems to be completely worn out. She has no appetite and absolutely no ambition for work, study or play. She does not lose flesh or grow thin at all, but her
